# Circulating Exosomal miR-181b-5p Promoted Cell Senescence and Inhibited Angiogenesis to Impair Diabetic Foot Ulcer via the Nuclear Factor Erythroid 2-Related Factor 2/Heme Oxygenase-1 Pathway

**DOI:** 10.3389/fcvm.2022.844047

**Published:** 2022-04-20

**Authors:** Shaohua Wang, Min Shi, Jing Zhou, Wenjing Wang, Yuanyuan Zhang, Yongjun Li

**Affiliations:** ^1^Hebei Key Laboratory of Laboratory Medicine, Department of Clinical Laboratory, The Second Hospital of Hebei Medical University, Shijiazhuang, China; ^2^Department of Endocrinology, The Second Hospital of Hebei Medical University, Shijiazhuang, China

**Keywords:** diabetic foot ulcer, exosomes, miR-181b-5p, angiogenesis, senescence (leaf)

## Abstract

Endothelial cell dysfunction is the main contributing factor of diabetic foot ulcer (DFU). Circulating exosomes have been found to play an important role in many processes, such as cell senescence and angiogenesis. However, the underlying roles and mechanism of circulating exosomes in the onset and progression of DFU remain unclear. In this study, we isolated exosomes from the plasma of patients with DFU (DFU-Exos) and non-diabetic foot wounds (NDF-Exos). DFU-Exos promoted cell senescence and inhibited tube formation in Human Umbilical Vein Endothelial Cells (HUVECs), unlike NDF-Exos. Several datasets suggest that miR-181b-5p expression might be enriched in exosomes from DFU; this was verified using quantitative real-time PCR (qRT-PCR). We also found that miR-181b-5p, which was taken up by HUVECs, promoted cell senescence and inhibited tube formation. Dual luciferase reporter assay, qRT-PCR, Western blotting, and immunofluorescence staining confirmed that miR-181b-5p could negatively regulate nuclear factor erythroid 2-related factor 2 (NRF2) expression by binding to its 3′ UTR, thus further suppressing heme oxygenase-1 (HO-1) expression. In addition, NRF2 and HO-1 inhibitors could also rescue the effects of senescence and tube formation exerted by miR-181b-5p inhibitor. *In vivo* experiments showed that exosomes isolated from HUVECs which inhibited miR-181b-5p expression promoted angiogenesis to further restore the capacity of wound healing. In conclusion, this study indicated that circulating exosomal miR-181b-5p promoted cell senescence and inhibited angiogenesis to impair wound healing in DFU by regulating the NRF2/HO-1 pathway.

## Introduction

Diabetic foot ulcer (DFU), which results from diabetes, is a serious chronic complication. The 5-year mortality of patients with DFU is much higher than that of patients without DFU (1). Although there are conventional and newly developed treatments for DFU, the outcomes are not satisfactory (2). Studies have shown that hyperglycemia-induced vascular damage and angiogenesis impairment are the major causes of DFU (3). A hyperglycemic environment is the proximal trigger for cell senescence, which leads to dysfunction of endothelial cells (ECs) and low levels of angiogenesis (4). However, the roles and underlying mechanism of blood hyperglycemic environment-induced cell senescence and angiogenesis impairment in the progression of DFU are unclear.

Exosomes are a group of small membranous vesicles that are secreted by almost all types of cells, including ECs (5). Increasing evidences indicate that exosomes can carry bioactive microRNA (miRNA), protein, and mRNA to recipient cells to change the cell phenotype (6). miRNAs are a group of small non-coding RNAs that have a length of approximately 22 nt. Evidences show that miRNA can activate or inhibit mRNA expression to participate in many processes (7). In addition, exosomes that carry miRNA participate in many pathological processes, including that of DFU. For example, exosomal miR-15a-3p expression is enriched both in the serum of patients with DFU and Human Umbilical Vein Endothelial Cells (HUVECs) under high glucose conditions, and the overexpression of miR-15a-3p impairs HUVECs functionality (8). Another report showed that miR-20b-5p expression is enriched in exosomes isolated from patients with T2DM. Knocking out miR-20b-5p accelerates wound healing by enhancing wound angiogenesis in cases of diabetes-associated impaired wound healing (9). Such evidence suggests that exosomes secreted by patients with DFU carry active miRNA to participate in the progression of DFU; this motivated us to investigate the mechanism of exosomal miRNA in the onset and progression of DFU.

In this study, we isolated exosomes from patients with DFU or non-diabetic foot wounds (NDF) and verified the differential miRNA expression among them to determine the roles and mechanism of exosomal miRNAs in the onset and progression of DFU.

## Materials and Methods

### Cell Culture and Transfection

Human umbilical vein endothelial cells (Cat. no. 8000; ScienCell Research Laboratories, Carlsbad, CA, United States) were cultured in ECM (Cat. no. 1001; ScienCell Research Laboratories) containing 5% fetal bovine serum (FBS; cat. no. 0025; ScienCell Research Laboratories), 1% penicillin/streptomycin (PS; cat. no. 0503; ScienCell Research Laboratories), and 1% endothelial growth factor (EGF; cat. no. 1052; ScienCell Research Laboratories). A293 cells (Fenghbio, Changsha, China) were cultured in Dulbecco’s modified Eagle medium (DMEM; 4.5 g/L D-glucose, HyClone, Logan, UT, United States) containing 10% FBS and 1% PS. All cells were cultured in a humidified incubator at 37°C with 5% CO_2_. ECM containing 2% FBS, 1% PS, and 1% EGF along with D-glucose (Sigma-Aldrich, St. Louis, MO, United States) was used to generate hyperglycemic conditions. D-mannitol (Sigma-Aldrich) was also used to induce a similar osmotic pressure. Lipofectamine 2000 (Invitrogen, Carlsbad, CA, United States) was used to transfect cells according to the manufacturer’s protocol. NRF2 inhibitor ML385 (3 μM) and HO-1 inhibitor ZnPP (10 μM) were used to incubate cells.

### Isolation of Exosomes

Exosomes from three diabetic foot ulcer patients’ plasma and three non-diabetes foot trauma patients’ plasma were considered as DFU-Exos and NDF-Exos. This experiment was approved by the Ethics Committee of The Second Hospital of Hebei Medical University. Exosomes from culture supernatant of HUVECs were grown in an exosome-free culture medium. Briefly, the plasma or culture medium was centrifuged at 2,000 × *g* for 30 min at 4°C. The supernatant was centrifuged at 10,000 × *g* in 4°C for 70 min and filtered through a 0.22 μm filter. Next, the supernatant was ultracentrifuged at 100,000 × *g* in 4°C for 70 min and the resulting liquid was discarded. Finally, the precipitate was washed with 5 mL PBS and ultracentrifuged (100,000 × *g*, 70 min) at 4°C. The precipitate contained the exosomes used to perform further experiments.

### Transmission Electron Microscopy and Nanoparticle Tracking Analysis

Twenty microliters of exosomes were used for TEM. The morphology of the exosomes was observed using a transmission electron microscope (HT-7700, Hitachi, Japan). NTA (N30E, NanoFCM, China) was used to analyze the size and concentration of exosomes.

### Exosome Labeling and Cellular Uptake

1,1′-dioctadecyl-3,3,3′,3′-tetramethylindocarbocyanine perchlorate (DiO; Sigma, St. Louis, MO, United States) was used to label exosomes according to the manufacturers’ protocol (10). DiO labeled the exosomes as our previous study described. The exosomes uptaken were imaged by a fluorescence microscope (IX73, Olympus, Japan).

### Quantitative Real-Time PCR

Total RNA was isolated from cells using TRIzol reagent (Invitrogen). Exosomal miRNA was extracted using an miRcute miRNA isolation kit (Tiangen Biotech, Beijing, China). The concentration of RNA was measured using a NanoDrop One (Thermo Fisher Scientific, Wilmington, DE, United States). cDNA was synthesized using a high-capacity cDNA reverse transcription kit (Thermo Fisher Scientific). qRT-PCR was performed via SYBR Green assays (Genecopoeia, Guangzhou, China) using a Bio-Rad system (Bio-Rad, Hercules, CA, United States). U6 or β-actin was used as the internal control to normalize miR-181b-5p or mRNA expression, respectively. The 2^–ΔΔCT^ formula was used to calculate the fold change of miRNA or mRNA expression. Table 1 showed the sequences of primers.

**TABLE 1:** Primers sequences used in this study.

miR-181b-5p RT	CTCAACTGGTGTCGTGGAGTCGGCAATT CAGTTGAGACCCACCGAC
miR-181b-5p F	ACACTCCAGCTGGGAACATTCATTGCTG
U6 F	CTCGCTTCGGCAGCACA
U6 R	AACGCTTCACGAATTTGCGT
Reverse	TGGTGTCGTGGAGTCG
NRF2 F	AGGTTGCCCACATTCCCAAA
NRF2 R	AATGTCTGCGCCAAAAGCTG
miR-181b-5p mimics	AACATTCATTGCTGTCGGTGGGT
miR-181b-5p inhibitor	ACCCACCGACAGCAATGAATGTT

### Dual Luciferase Assay

A293 cells were seeded in a six-well plate and co-transfected with pmiR-GLO-NRF2 3′ UTR wild-type (pmiR-GLO-NRF2 3′ UTR wt) or mutation (pmiR-GLO-NRF2 3′ UTR mut) and miR-181b-5p mimics. After 48 h, the cells were lysed using RIPA (Solarbio, China). A Dual Luciferase Assay System (Promega, Madison, WI, United States) was used to measure the luciferase activity using a Flash and Glow reader.

### Immunofluorescence Staining

Human umbilical vein endothelial cells or tissue slices were treated with 4% paraformaldehyde, permeabilized in 0.2% Triton X-100 for 10 min, and blocked with goat serum for 1 h at room temperature. The cells or tissue slices were incubated overnight with primary antibodies of NRF2 (1:50; 66504-1-lg, Proteintech, China) or CD31 (1:50; AF6191, Affinity, China). They were then incubated with secondary antibodies (1:50; Affinity) after washing with PBS. Nuclei were stained with DAPI (Solarbio) and imaged using a fluorescence microscope (IX73, Olympus, Japan).

### Immunohistochemical Staining

Immunohistochemical staining was performed according to the manufacturer’s protocol (ZSBio, China). After dewaxing and hydration, paraffin sections were immersed in endogenous peroxidase blocker at room temperature for 10 min and then washed with PBS. The sections were incubated with CD31 (1:50; AF6191, Affinity) at 37°C for 60 min and then washed with PBS. An enhancer (100 μL) was added for 20 min and then the sections were again washed with PBS. Next, the sections were incubated with enzyme-labeled goat anti-rabbit IgG polymer for 20 min and then washed with PBS. The labeling was visualized using DAB (ZSBio) and hematoxylin (Solarbio) and captured using a microscope (IX73).

### Cell Viability (CCK-8 Assay)

We seeded 3000 cells per well in a 96-well plate after transfection or incubation with exosomes. After 24 h, 10 μL of CCK-8 (Solarbio) solution was added to the medium. The OD value was measured at 450 nm using a microplate reader (MD2, Molecular Devices, United States).

### β-Galactosidase Staining

β-galactosidase staining was performed according to the manufacturer’s protocol (Beyotime, China). The cells were seeded in a six-well plate and incubated with 1 mL of β-galactosidase staining fixation fluid for 15 min at room temperature after washing the cells with PBS. After that, the fixation fluid was removed and the cells were incubated with 1 mL staining working solution which was composed of 10 μL staining solution A, 10 μL staining solution B, 10 μL staining solution C and 50 μL X-Gal solution. The cells were incubated at 37°C overnight and captured the images using a microscope (IX73).

### Tube Formation

Matrigel was coated in a 24-well plate in a humidified incubator at 37°C for 2 h. After incubation with exosomes or transfection, HUVECs (50,000 cells per well) were seeded into the plate. After 24 h, tube formation was imaged using a microscope (IX73).

### Diabetic Foot Ulcer Mouse Skin Wound Model

C57BL/6J mice (male, 6 weeks old, weighing 20–30 g, SPF-Tsinghua, China) were used to establish a DFU mouse skin wound model. The mice were randomly separated into three groups with 5 mice each group. Mice in the normal group were injected with saline under normal feeding. Mice in the diabetes group were fed a high-glucose and high-fat diet and intraperitoneally injected with streptozotocin (STZ; 45 mg/kg, Sigma-Aldrich, St. Louis, MO, United States). After 48 h, the blood glucose level was measured using a blood glucose monitoring system (Roche, Switzerland). Blood glucose levels ≥16.7 mM continuously for 10 days were considered characteristic of the diabetes model. Two weeks later, a full-thickness excisional skin wound was inflicted on the upper backs of the mice (11, 12). The mice were then randomly divided into three groups with 5 mice each group: normal control group was C57BL/6J mice injected with exosomes from HUVECs transfected with ASO-NC (NC group), diabetes group was diabetic mice injected with exosomes from HUVECs transfected with ASO-NC (DM + Exo-NC), and Exo-miR-181b-5p inhibitor group was diabetic mice injected with exosomes from HUVECs transfected with miR-181b-5p inhibitor (DM + Exo-miR-181b-5p inhibitor). On days 1, 4, 7, 11, and 14 post wounding, the wounds were photographed and measured using a ruler. The diameter was the average of maximum diameter and minimum diameter. All the animal experiments were conducted in accordance with the National Institute of Health guidelines (NIH Publication No. 8023, revised 1978) and were approved by the Research Ethics Committee of The Second Hospital of Hebei Medical University.

### Western Blotting

The proteins isolated from exosomes or cells were homogenized in RIPA buffer (Solarbio) and the protein concentration was measured using a BCA protein assay kit (Beyotime, China). The proteins were separated by SDS-PAGE and transferred to PVDF membranes (Millipore, Burlington, MA, United States). The membranes were incubated in BSA (Solarbio) and then with the antibody TSG101 (67381-1-lg, Proteintech), CD63 (67605-1-lg, Proteintech), Calnexin (Cat No. 10427-2-AP, Proteintech), PCNA (610664, BD, United States), VEGF (2E2H9, Proteintech), p16^INK4A^ (10883-1-AP, Proteintech), or NRF2 (66504-1-lg, Proteintech) overnight. Secondary antibodies (5230-0336 and 5230-0341, KPL, IN, United States) were added 1 h after washing the PVDF membranes with TBST (Solarbio). Bands were developed using ECL solution (Solarbio) and imaged using a ChemiDoc MP (Bio-Rad).

### Statistical Analysis

All statistical analyses were performed using GraphPad Prism software (Version 5.0; GraphPad Software, Inc., San Diego, CA, United States). The data was expressed as means ± standard deviations. Statistical differences between two groups were determined using Student’s *t*-test. Statistical differences >2 groups were determined using One-way ANOVA or two-way ANOVA followed by Tukey’s *post hoc* test. All data were tested for normality and equal variance. Statistical significance was set at *p* ≤ 0.05.

## Results

### Exosomes Secreted Into Plasma of Patients With Diabetic Foot Ulcer Promoted Cell Senescence and Inhibited Tube Formation in Human Umbilical Vein Endothelial Cells

**FIGURE 1:**
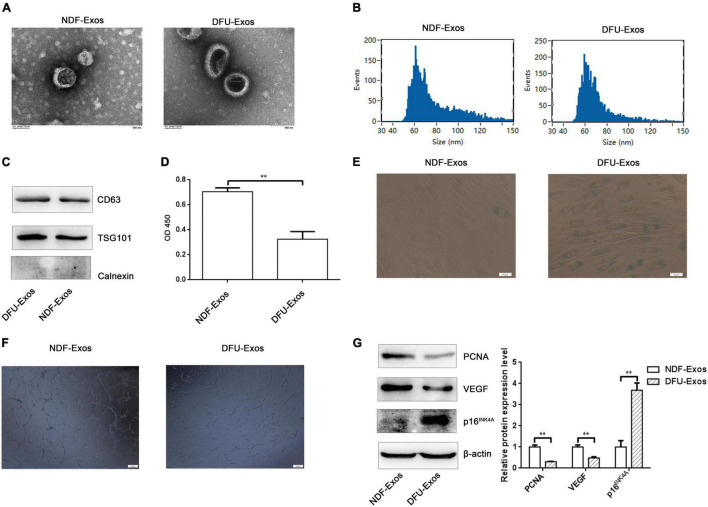
Exosomes secreted into the plasma of patients with DFU promoted cell senescence and inhibited tube formation in HUVECs. (A) TEM image showing the shape and sides of exosomes isolated from NDF-Exos and DFU-Exos. (B) NTA results showing the size of the exosomes. (C) Western blotting showing the expression of CD63, TSG101, and Calnexin in the exosomes. (D) Results of CCK-8 assay to detect the effects of NDF-Exos and DFU-Exos on cell viability. **P < 0.01 NDF-Exos vs. DFU-Exos. (E) β-gal stain showing β-galactosidase activity in HUVECs treated with NDF-Exos and DFU-Exos. (F) Tube formation in NDF-Exos and DFU-Exos groups. (G) Western blotting showing the expression of PCNA, VEGF, and p16^INK4A^ in HUVECs incubated with exosomes. **P < 0.01 DFU-Exos vs. NDF-Exos.

Plasma exosomes of patients with non-diabetic foot wound (NDF-Exos) and DFU (DFU-Exos) were isolated by ultracentrifugation. NDF-Exos and DFU-Exos were observed using TEM (Figure 1A). NTA showed that the diameters of both DFU-Exos and NDF-Exos groups are in 40–150 nm. The median diameters of NDF-Exos and DFU-Exos were 70.25 and 66.25 nm (Figure 1B). Western blotting showed that exosome-associated markers (CD63 and TSG101) were positively expressed and Calnexin was negatively expressed (Figure 1C). We then assessed the effects of NDF-Exos or DFU-Exos on HUVECs. CCK-8 assay indicated that cell viability decreased when HUVECs were incubated with DFU-Exos (Figure 1D). DFU-Exos promoted cell senescence compared with the NDF-Exos group; this was detected using the senescence-associated β-galactosidase activity in HUVECs (SA-β-Gal) (Figure 1E). Moreover, tube formation reduced in HUVECs treated with DFU-Exos (Figure 1F). Western blotting showed that PCNA and VEGF expression decreased, whereas p16^INK4A^ expression increased, in DFU-Exos-treated HUVECs compared with that in NDF-Exos-treated cells (Figure 1G).

### Exosomal miR-181b-5p From DFU-Exos Promoted Cell Senescence and Inhibited Tube Formation in Human Umbilical Vein Endothelial Cells

**FIGURE 2:**
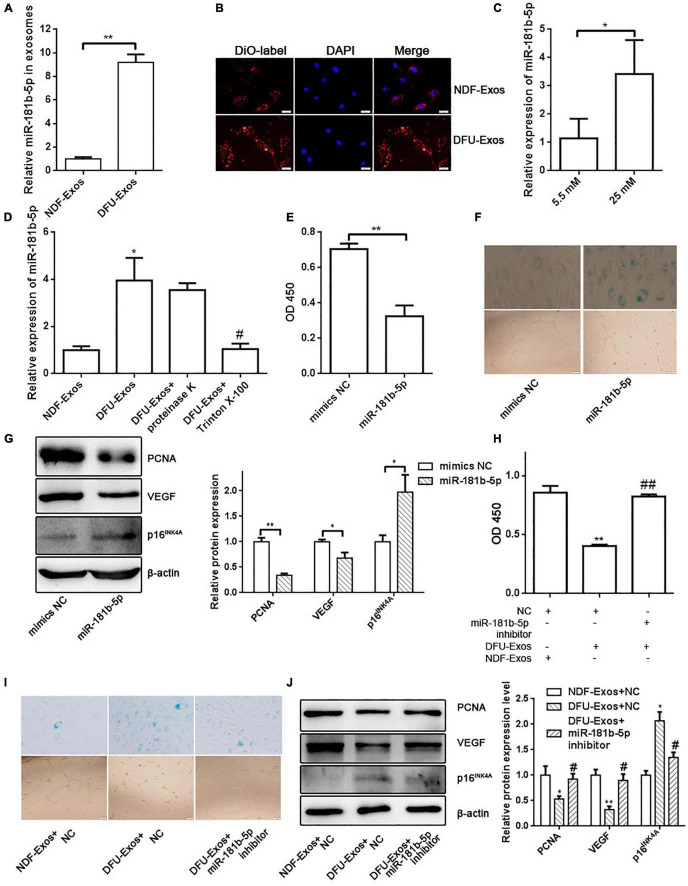
Exosomal miR-181b-5p from DFU-Exos promoted cell senescence and inhibited tube formation in HUVECs. (A) miR-181b-5p expression in NDF-Exos and DFU-Exos detected by qRT-PCR. **P < 0.01 DFU-Exos vs. NDF-Exos. (B) DiO-labeled showing the uptake of exosomes by HUVECs. (C) miR-181b-5p level in exosomes isolated from HUVECs cells grown in culture medium containing 5.5 mM or 25 mM glucose. *P < 0.05 5.5 mM vs. 25 mM. (D) miR-181b-5p level in HUVECs was detected by qRT-PCR. *P < 0.05 vs. NDF-Exos, ^#^*P* < 0.05 vs. DFU-Exos. CCK-8 assay (E), β-gal staining and tube formation (F) were used to detect the effects of miR-181b-5p on cell viability, cell senescence and tube formation. **P < 0.01 miR-181b-5p vs. mimics NC. (G) Western blotting showing the expression of PCNA, VEGF, and p16^INK4A^ in HUVECs overexpressing miR-181b-5p. *P < 0.05, **P < 0.01 miR-181b-5p vs. mimics NC. CCK-8 assay (H), β-gal staining and tube formation (I) showing cell viability, senescence and tube formation in rescue experiment. **P < 0.01 vs. NC + NDF-Exos, ^##^*P* < 0.01 vs. NC + DFU-Exos. (J) Western blotting showing the expression of PCNA, VEGF, and p16^INK4A^ in HUVECs. *P < 0.05, **P < 0.01 vs. NDF-Exos + NC, ^#^*P* < 0.05 vs. DFU-Exos + NC.

To determine the molecular mechanism by which DFU-Exos impaired HUVECs functionality, we retrieved a dataset from the Gene Expression Omnibus (GEO) of the National Center for Biotechnology Information and found that exosomal miR-181b-5p expression was elevated in patients with DFU compared with that in patients with NDF or healthy people (13). qRT-PCR verified the higher level of expression of miR-181b-5p in exosomes from the plasma of patients with DFU (Figure 2A). DiO-labeled DFU-Exos or NDF-Exos showed the uptake of exosomes by HUVECs (Figure 2B). Furthermore, exosomes from HUVECs grown with a high concentration of glucose (25 mM) exhibited higher levels of miR-181b-5p than those from cells grown with normal glucose (5.5 mM) levels (Figure 2C). In addition, the level of miR-181b-5p was higher in HUVECs which were incubated with DFU-Exos than those incubated with NDF-Exos (Figure 2D). Because the miRNAs might be attached outside the exosomes, we treated exosomes with proteinase K and found that proteinase K did not affect the miR-181b-5p level in HUVECs which were incubated with DFU-Exos (Figure 2D). In contrast, treatment with the phospholipid membrane disruptor Triton X-100 led to a degradation of miR-181b-5p which indicated that the high level of miR-181b-5p is from the DFU-Exos (Figure 2D). Overexpression of miR-181b-5p by transfecting miR-181b-5p mimics in HUVECs inhibited cell viability and promoted cell senescence (Figures 2E,F). We also found that miR-181b-5p could inhibit tube formation in HUVECs compared with the control group (Figure 2F). In addition, Western blotting showed the PCNA and VEGF expression decreased, whereas p16^INK4A^ expression increased, when miR-181b-5p was overexpressed in HUVECs (Figure 2G). To further evaluate the roles of miR-181b-5p carried by DFU-Exos on cell senescence and tube formation of HUVECs, we performed a rescue experiment. Inhibition of miR-181b-5p by miR-181b-5p inhibitor could partly rescue the effects of cell viability, senescence and tube formation exerted by DFU-Exos (Figures 2H,I). Nevertheless, the expression of PCNA, VEGF, and p16^INK4A^ in HUVECs incubated with DFU-Exos also partly rescued by inhibiting miR-181b-5p expression (Figure 2J).

### miR-181b-5p Regulated Nuclear Factor Erythroid 2-Related Factor 2 Expression by Targeting Its 3′ UTR

**FIGURE 3:**
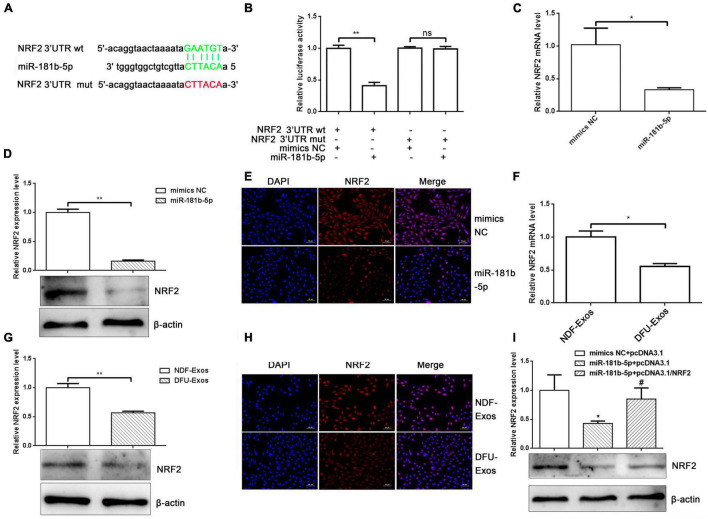
miR-181b-5p regulated NRF2 expression by targeting its 3′ UTR. (A) Binding of miR-181b-5p and NRF2 3′ UTR. (B) Dual luciferase assay showing luciferase activity alteration upon co-transfecting pmiR-GLO-NRF2 3′ UTR wild-type or mutated and overexpressing or inhibiting miR-181b-5p. **P < 0.01 vs. mimics NC + NRF2 3′ UTR wt. (C) qRT-PCR, (D) Western blotting, and (E) immunofluorescence stain showing the expression of NRF2 after overexpressing miR-181b-5p. *P < 0.05, **P < 0.01 vs. mimics NC. (F) qRT-PCR results, (G) Western blotting, and (H) immunofluorescence stain showing the expression of NRF2 in HUVECs incubated with NDF-Exos and DFU-Exos. *P < 0.05, **P < 0.01 vs. NDF-Exos. Western blotting (I) showed the expression of HO-1 in rescue experiments. *P < 0.05 vs. mimics NC + pcDNA3.1, ^#^*P* < 0.05 vs. miR-181b-5p + pcDNA3.1.

It is well known that miRNAs carry out their functions by binding to target mRNA 3′ UTRs. We used Starbase^1^ to predict miR-181b-5p targets and found that NRF2 might be one (Figure 3A). The binding sites are shown in Figure 3A. To verify their relationship, a dual luciferase reporter assay was performed. We cloned a NRF2 3′ UTR fragment (wild-type or mutation) containing binding sites or mutation for miR-181b-5p into pmiR-GLO (pmiR-GLO-NRF2 3′ UTR wt or mut). Cells co-transfected with pmiR-GLO-NRF2 3′ UTR wt and miR-181b-5p mimics inhibited luciferase activity, unlike cells co-transfected with pmiR-GLO-NRF2 3′ UTR wt and NC mimics. No effects on luciferase activity were observed when pmiR-GLO-NRF2 3′ UTR mut was transfected into A293 cells along with miR-181b-5p mimics (Figure 3B). To further identify the effects of miR-181b-5p on NRF2 mRNA and protein expression, we transfected miR-181b-5p or NC mimics into HUVECs. qRT-PCR, Western blotting, and immunofluorescence staining showed that miR-181b-5p overexpression decreased the mRNA and protein levels of NRF2 (Figures 3C–E). In addition, DFU-Exos reduced the mRNA and protein levels of NRF2 (Figures 3F–H). More importantly, overexpression of NRF2 partly rescued the inhibition of NRF2 induced by miR-181b-5p (Figure 3I). These results indicated that exosomal miR-181b-5p inhibited NRF2 expression by targeting its 3′ UTR.

### Nuclear Factor Erythroid 2-Related Factor 2 and Its Downstream Gene Heme Oxygenase-1 Inhibited Cell Senescence and Promoted Tube Formation in Human Umbilical Vein Endothelial Cells

**FIGURE 4:**
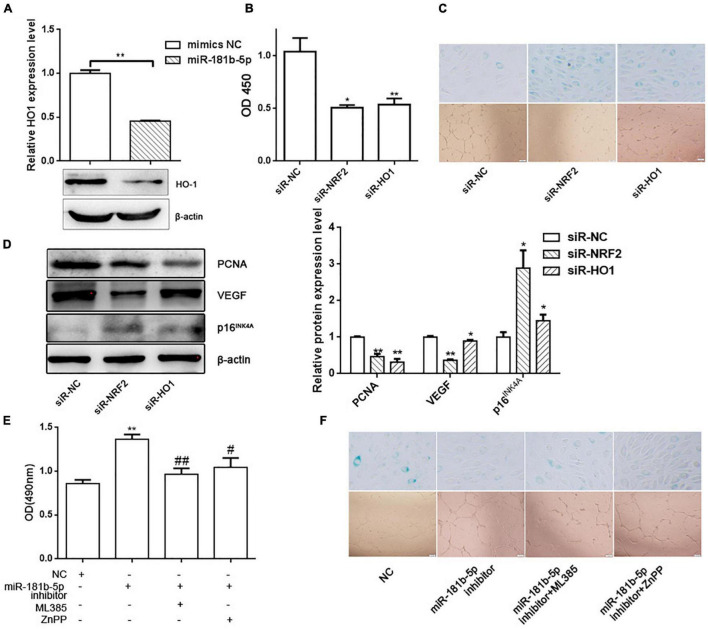
NRF2 and HO-1 inhibited cell senescence and promoted tube formation in HUVECs. (A) Western blotting showing the expression of HO-1 after overexpressing miR-181b-5p. **P < 0.01 vs. mimics NC. CCK-8 assay (B), β-gal staining and tube formation (C) showing the effects of siR-NRF2 or siR-HO-1 on cell viability, cell senescence and tube formation in HUVECs. *P < 0.05, **P < 0.01 vs. siR-NC. (D) Western blotting showing the PCNA, VEGF and p16^INK4A^ expression in rescue experiments. *P < 0.05, **P < 0.01 vs. siR-NC. (E) CCK-8 assay showing cell viability in rescue experiments. **P < 0.01 vs. NC, ^#^*P* < 0.05, ^##^*P* < 0.01 vs. miR-181b-5p inhibitor. (F) β-gal staining and tube formation showing cell senescence and tube formation ability in rescue experiments.

Considering that HO-1 gene is the downstream gene of NRF2, we also verified that overexpression of miR-181b-5p inhibited HO-1 expression in HUVECs (Figure 4A). To further identify whether NRF2/HO-1 pathway is involved in the HUVECs senescence and tube formation, we knocked down NRF2 or HO-1 expression by siRNA. CCK-8, β-galactosidase activity and tube formation were performed, which indicated that knock down of NRF2 or HO-1 expression promoted cell senescence, and inhibited cell activity and tube formation in HUVECs (Figures 4B,C). Western blotting showed that the PCNA and VEGF expression decreased, whereas p16^INK4A^ expression increased, when NRF2 or HO-1 was knocked down in HUVECs (Figure 4D). In addition, NRF2 and HO-1 inhibitors, ML385 and ZnPP, could also rescue the effects on cell activity, senescence and tube formation exerted by miR-181b-5p inhibitor (Figures 4E,F).

### Exosomes Isolating From Human Umbilical Vein Endothelial Cells Which Inhibited miR-181b-5p Expression Restored the Capacity of Wound Healing

**FIGURE 5:**
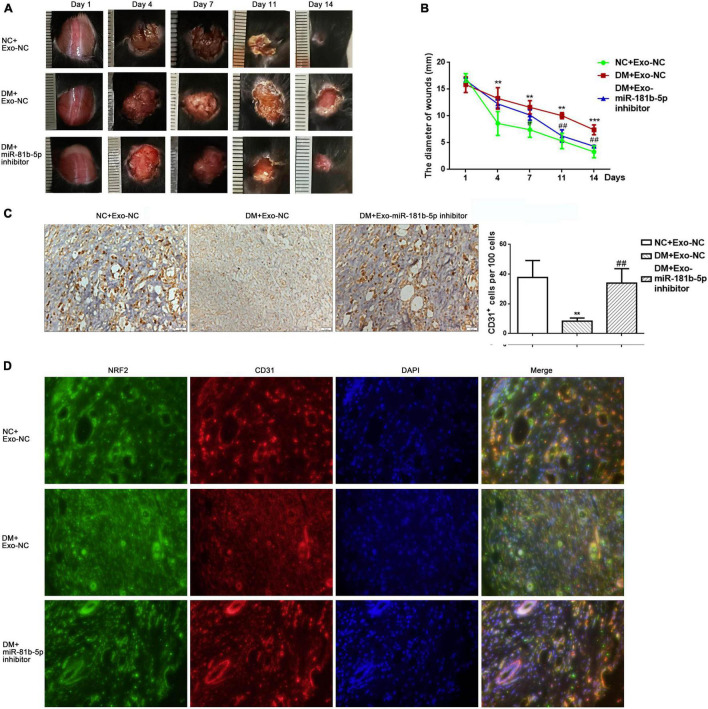
Knockdown of miR-181b-5p in exosomes restored wound healing. (A,B) Wound closure rate of full-thickness dermal defects on days 1, 4, 7, 11, and 14 after skin operation. **P < 0.01, ***P < 0.001 vs. NC + Exo-NC, ^#^*P* < 0.05, ^##^*P* < 0.01 vs. DM + Exo-NC. (C) CD31 immunohistochemical stain showing the vascular density in three groups. **P < 0.01 vs. NC + Exo-NC, ^##^*P* < 0.01 vs. DM + Exo-NC. (D) Immunofluorescence stain showing the expression of NRF2 in a CD31-positive area.

To further confirm the effects of exosomal miR-181b-5p on wound repair *in vivo*, full-thickness cutaneous wounds were generated on the backs of mice. Equal volumes of exosomes isolating from culture medium of HUVECs transfected with inhibitor NC (Exo-NC) or miR-181b-5p inhibitor (Exo-miR-181b-5p inhibitor) were injected around the wounds. The disappearance of wounds showed that the Exo-miR-181b-5p inhibitor accelerated wound healing in the diabetes mice group compared with that in the DM + Exo-NC group (Figures 5A,B). CD31 immunohistochemical staining indicated that the CD31^+^ area was higher in the DM + Exo-miR-181b-5p inhibitor group than in the DM + Exo-NC group which indicated that Exo-miR-181b-5p inhibitor group showed higher vascular density than DM + Exo-NC group (Figures 5C,D). Immunofluorescence staining showed that the expression of NRF2 in ECs was higher in the DM + Exo-miR-181b-5p inhibitor group than in the DM + Exo-NC group (Figure 5D). These results indicated that exosomal miR-181b-5p could impair wound healing by inhibiting angiogenesis *in vivo*.

## Discussion

Endothelial cells senescence and insufficient angiogenesis are associated with the onset and progression of DFU (3, 14). The development of efficient approaches to delay senescence and restore angiogenesis would be beneficial to DFU treatment. Evidence shows that circulating exosomes influence EC senescence and angiogenesis (15). The main findings of this study are as follows: (i) exosomes isolated from the plasma of patients with DFU carried high levels of miR-181b-5p compared with those from patients without NDF; (ii) miR-181b-5p promoted cell senescence and impaired tube formation in HUVECs; and (iii) miR-181b-5p suppressed NRF2 expression by binding to the NRF2 mRNA 3′ UTR, which in turn inhibited HO-1 expression.

Exosomes, which are abundant in whole blood and culture media, have been considered as ideal carriers for the treatment of many diseases (16). They not only have the ability to carry active molecular contents but also perform functions in the progression of DFU. Several studies have shown that exosomes circulate in plasma from patients with DFU. Exosomes from DFU patients impaired HUVECs angiogenesis *in vitro* and suppressed cutaneous wound repair *in vivo* (8). Hyperglycemia triggers cell senescence and changes the phenotype of HUVECs. In this study, we focused on the roles and mechanism of circulating exosomes in the onset and progression of DFU. We found that DFU-Exos promoted cell senescence and inhibited angiogenesis in HUVECs. DFU-Exos inhibited the expression of the proliferation-related protein, PCNA, and the angiogenesis maker, VEGF, whereas it promoted the expression of the cell senescence marker, p16^INK4A^. Moreover, labeled DFU-Exos or NDF-Exos could be uptaken by HUVECs, thus impairing HUVECs functions.

Differentially expressed miRNA in exosomes have been shown to be related to the onset and progression of DFU. For example, exosomal miR-24-3p levels are elevated in patients with diabetes and knocking down miR-24-3p accelerates wound healing (17). Based on the dataset from different miRNA microarrays, we isolated exosomes from the plasma of patients with DFU or NDF and found that miR-181b-5p was more enriched in patients with DFU than in those with NDF. Similarly, the expression of miR-181b-5p was higher in exosomes under high-glucose conditions than under normal glucose conditions. Overexpression of miR-181b-5p could promote cell senescence and reduce tube formation *in vitro*, and inhibition of miR-181b-5p could partly rescue the effects on cell viability, senescence and tube formation exerted by DFU-Exos, which suggested that miR-181b-5p was enriched in DFU-Exos and impaired HUVECs functions. Next, we inflicted full-thickness cutaneous wounds on the backs of diabetic and normal mice. Exosomes isolated from HUVECs inhibiting miR-181b-5p expression were injected around the wounds. These exosomes exhibited a higher density of vessels, both macrovascular and microvascular, in wound tissues. These results indicated that treatment the wound with exosomes isolated from HUVECs which inhibited the high level of miR-181b-5p might be helpful to the recovery of DFU.

Through bioinformatics analysis, we found that NRF2 3′ UTR has binding sites for miR-181b-5p. A dual luciferase assay verified that miR-181b-5p could bind to NRF2 3′ UTR directly. Overexpression of miR-181b-5p inhibited NRF2 mRNA and protein levels in HUVECs. In addition, exosomes from patients with DFU, which were enriched with miR-181b-5p, reduced NRF2 mRNA and protein levels. Moreover, overexpression of NRF2 could rescue the inhibition of NRF2 mRNA and protein level induced by miR-181b-5p. *In vivo* experiments also showed that miR-181b-5p inhibition promoted NRF2 expression in ECs. NRF2 belongs to the Cap-n-Collar family of basic leucine zipper proteins that regulates the outcomes of oxidative stress and senescence (18). Oxidative stress is related to vascular aging (19). NRF2 dysfunction triggers cell senescence, as knockout of NRF2 elevates the expression of the senescence markers p16^INK4A^ and p21 (20). Knockdown of NRF2 by siRNA impairs angiogenesis of ECs (21). High levels of NRF2 in adipose stem cell-derived exosomes can promote angiogenesis in endothelial progenitor cells and protect cells (14) against stresses in DFU. The HO-1 gene is the downstream gene of NRF2. The NRF2/HO-1 pathway plays a protective role in the process of cell senescence (22). In this study, we found that overexpression of miR-181b-5p inhibited the expression of HO-1 in HUVECs, which indicated that HO-1 participated in the miR-181b-5p/NRF2 regulatory axis. Knockdown of NRF2 or HO-1 expression, as well as their inhibitors, induced a promotion of cell senescence and a inhibition of tube formation in HUVECs. These findings provided a novel insight into the circulating exosomes induced HUVECs function impairment in DFU.

It is reasonable to conclude that miR-181b-5p enriched in circulating exosomes in patients with DFU attenuates the expression of NRF2 and thus promotes cell senescence and impairs angiogenesis in DFU. This newly identified circulating exosomal miR-181b-5p may be a potential target for the recovery of DFU.

## Data Availability Statement

The original contributions presented in the study are included in the article/Supplementary Material.

## Ethics Statement

The studies involving human participants were reviewed and approved by the Research Ethics Committee of The Second Hospital of Hebei Medical University. Written informed consent to participate in this study was provided by the participants’ legal guardian/next of kin. The animal study was reviewed and approved by the Research Ethics Committee of The Second Hospital of Hebei Medical University.

## Author Contributions

YL and SW designed the experiments and wrote the manuscript. SW, YZ, and WW carried out the data analyses and conducted the experiments. JZ performed the experiments *in vivo*. MS prepared the figures. All authors read and approved the final submitted manuscript, and agreed to be accountable for the content of the work.

## Conflict of Interest

The authors declare that the research was conducted in the absence of any commercial or financial relationships that could be construed as a potential conflict of interest.

## Publisher’s Note

All claims expressed in this article are solely those of the authors and do not necessarily represent those of their affiliated organizations, or those of the publisher, the editors and the reviewers. Any product that may be evaluated in this article, or claim that may be made by its manufacturer, is not guaranteed or endorsed by the publisher.

## Footnotes

1
https://starbase.sysu.edu.cn/
