# Supplementary figures and images for: Circulating Exosomal miR-181b-5p Promoted Cell Senescence and Inhibited Angiogenesis to Impair Diabetic Foot Ulcer via the Nuclear Factor Erythroid 2-Related Factor 2/Heme Oxygenase-1 Pathway
Source: Front Cardiovasc Med. 2022 Apr 20;9:844047. doi: 10.3389/fcvm.2022.844047 (PMC9067436; doi:10.3389/fcvm.2022.844047)

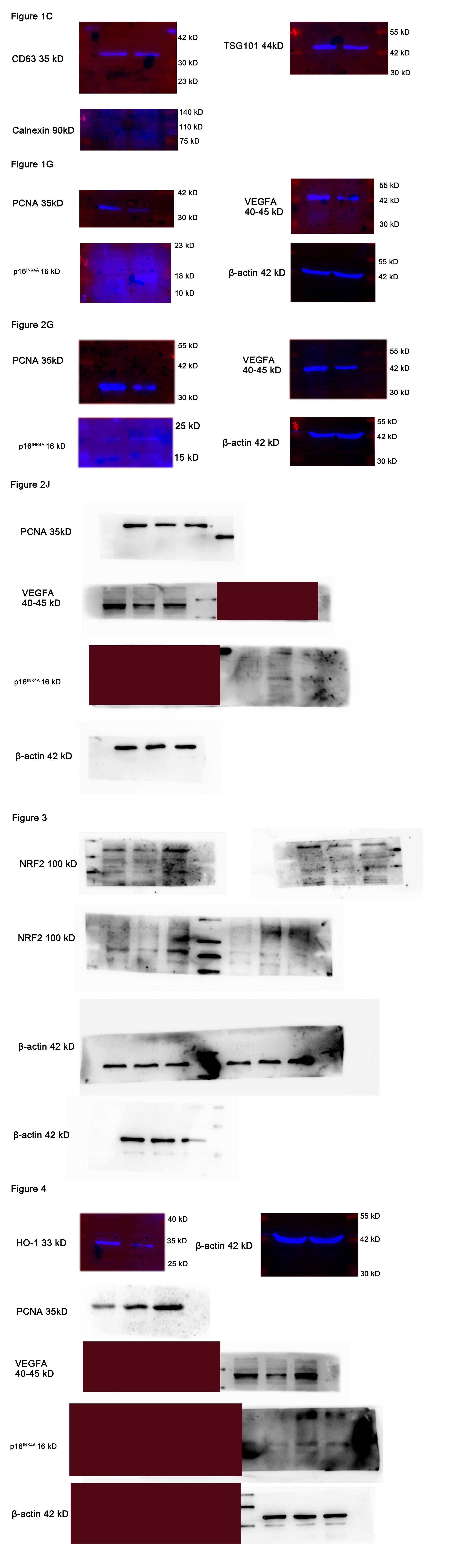

Supplement: Supplementary file 2 [file Image_1.jpg]
